# Correlation between antibiotic consumption and the occurrence of multidrug-resistant organisms in a Malaysian tertiary hospital: a 3-year observational study

**DOI:** 10.1038/s41598-022-07142-2

**Published:** 2022-02-24

**Authors:** Sin Yee Tan, Rahela Ambaras Khan, Khairil Erwan Khalid, Chun Wie Chong, Athirah Bakhtiar

**Affiliations:** 1 Pharmacy Department, Hospital Kuala Lumpur, Ministry of Health, Kuala Lumpur, Malaysia; 2 Medical Department, Hospital Kuala Lumpur, Ministry of Health, Kuala Lumpur, Malaysia; 3 School of Pharmacy, Monash University Malaysia, Bandar Sunway, Selangor, Malaysia

**Keywords:** Antibiotics, Antimicrobial resistance

## Abstract

Inappropriate use of antibiotics has been shown to contribute to the occurrence of multidrug-resistant organisms (MROs). A surveillance study was performed in the largest tertiary care hospital in Kuala Lumpur, Malaysia, from 2018 to 2020 to observe the trends of broad-spectrum antibiotics (beta-lactam/beta-lactamases inhibitors (BL/BLI), extended-spectrum cephalosporins (ESC), and fluoroquinolones (FQ)) and antibiotics against MRO (carbapenems, polymyxins, and glycopeptides) usage and the correlation between antibiotic consumption and MROs. The correlation between 3-year trends of antibiotic consumption (defined daily dose (DDD)/100 admissions) with MRO infection cases (per 100 admissions) was determined using a Jonckheere-Terpstra test and a Pearson’s Correlation coefficient. The antimicrobial resistance trend demonstrated a positive correlation between ESC and FQ towards the development of methicillin-resistant *Staphylococcus aureus* (MRSA), extended-spectrum beta-lactamase (ESBL)-producing *Klebsiella* spp, ESBL-producing *Escherichia coli (E. coli),* and MRO *Acinetobacter baumannii (A. baumannii)*. Increasing carbapenem consumption was positively correlated with the occurrence of ESBL-producing *Klebsiella spp* and *E. coli*. Polymyxin use was positively correlated with ESBL-producing *Klebsiella spp*, MRO *A. baumannii,* and carbapenem-resistant Enterobacteriaceae. The findings reinforced concerns regarding the association between MRO development, especially with a surge in ESC and FQ consumption. Stricter use of antimicrobials is thus crucial to minimise the risk of emerging resistant organisms.

## Introduction

The discovery of antibiotics in the early twentieth century has revolutionised infection control by saving countless human lives from bacterial infections. Nonetheless, pathogenic bacteria have since evolved and developed various resistance mechanisms against the antibiotics they were once susceptible to, leading to multidrug-resistant organisms (MROs)^1^. In 2014, the World Health Organization (WHO) reported an alarming rate of resistance to multiple classes of antibiotics in common community-acquired and healthcare-associated infections such as *Klebsiella pneumoniae*, *Escherichia coli* and *Staphylococcus aureus*^2^. Once a pathogen acquires resistance towards antibiotics, the efficacy of treatment decreases substantially, limiting the therapeutic options and complicating the treatment outcomes. Inevitably, MRO infections are often associated with higher mortality rates^3^. Studies have reported that antibiotic resistance is a serious predicament, especially in tertiary care settings^4^. Patients are more susceptible to severe nosocomial infections, leading to poorer clinical outcomes, longer hospital stays, and higher health expenditure^5–7^. However, despite the rise in antibiotic resistance, there has been a downturn in the evolution of new antibiotics since the 1990s, primarily due to regulatory barriers, technical difficulties and low profitability compared to long-term medications for chronic conditions^8–10^. If this remains without any intervention, infectious diseases that were successfully eradicated in the past may resurface.

Considering all the factors leading to antibiotic resistance, implementing antimicrobial stewardship programs (ASPs) is crucial to promote the judicious use of antibiotics, thus reducing antibiotic resistance. As such, periodic surveillance of antibiotic use and resistance at the local and national levels is vital to evaluate the effectiveness of ASPs and devise new strategies to address the gaps in the existing practices^11,12^. Although the Malaysian national data on antibiotic use and susceptibility/resistance patterns can be found in the annual National Surveillance of Antibiotic Resistance reports, Malaysian Statistics of Medicine, and National Antibiotic Guidelines (NAG), the antibiotic consumption and susceptibility profiles may vary in different localities ^13,14^. There is also a lack of evidence available in Malaysia that highlights the influence of broad-spectrum antibiotic usage toward the occurrence of MROs.

To address these existing gaps, the present study evaluated the 3-year trend of broad-spectrum antibiotics and antibiotics against MRO usage in Hospital Kuala Lumpur (HKL) and its influence on the occurrence of MROs. The main objectives of this study are to: (1) evaluate the trend of use of broad-spectrum antibiotics, (2) analyse the trend of occurrence of MROs and (3) determine the influence of broad-spectrum antibiotic usage on the occurrence of MROs. It was hypothesised that an increase in the trend of broad-spectrum antibiotics usage correlates with a surge in the occurrence of MROs.

## Materials and methods

### Study design and period

Quantitative research was conducted to analyse the three-year trend of systemic broad-spectrum antibiotics use and its influence on the MRO infection rates in HKL, a tertiary care hospital in Kuala Lumpur, Malaysia. It has 73 wards and 1570 beds with 19 clinical departments. Data on antibiotic consumption (expressed through DDD/100 admissions) was obtained from the pharmacy department, while MRO data were retrieved from the infection control department. The protocol was approved by the Medical Research Ethics Committee (MREC), Ministry of Health, Malaysia (NMRR-20-787-54141) and the Clinical Research Committee of HKL and in compliance with ethical principles outlined in the Declaration of Helsinki and Malaysian Good Clinical Practice (GCP) Guideline. The requirement for written informed consent from patients was waived by MREC due to the retrospective nature of the study. Furthermore, precautions were taken to ensure that all data and records confidentiality were only used for this study.

### Study definition

#### Antibiotic

Six groups of antibiotics that are classified under J01 (antibacterial for systemic use) according to the Anatomical Therapeutic Chemical (ATC) Classification System were included in this study. Broad-spectrum antibiotics selected for this study include amoxicillin/clavulanate, ampicillin/sulbactam, piperacillin/tazobactam, ceftriaxone, cefotaxime, ceftazidime, cefoperazone, cefoperazone/sulbactam, cefepime, ciprofloxacin and levofloxacin. Meanwhile, antibiotics against multidrug-resistant organisms (MROs) comprised of imipenem, meropenem, ertapenem, colistin, polymyxin B and vancomycin. Extended-spectrum cephalosporin (ESC) are cephalosporins with better activity against gram-negative microbes than first-generation agents. The following formula was used to calculate the defined daily dose (DDD) per 100 admissions (expressed as DDD/100 admissions) ^15^:

$${\text{Number of DDD per year }} = \frac{{{\text{Total antibiotic usage }}\left( {\text{g}} \right){\text{ for in}} - {\text{patient adults in a year}}}}{{{\text{DDD }}\left( {\text{from WHO}} \right)}}$$

$${\text{Number of DDD per 1}}00{\text{ admissions }} = \frac{{\text{Number of DDD per year}}}{{\text{Total admission for the particular year}}} \times { 1}00$$

#### Rate of MRO infections

The types of MROs analysed in this study were Methicillin-resistant *Staphylococcus aureus* (MRSA), extended-spectrum beta-lactamase (ESBL)-producing *Klebsiella spp*, ESBL-producing *Escherichia coli*, multidrug-resistant organisms (MRO) *Acinetobacter baumannii*, carbapenem-resistant Enterobacteriaceae (CRE) and vancomycin-resistant enterococci (VRE). The MRO cases were followed up by Infection Control Team through discussions with primary prescribers to differentiate between infection and coloniser; colonisers data were then excluded from this study. The rate of MRO infection per 100 admissions was calculated using the following formula:

$${\text{MRO infection cases per 1}}00{\text{ admissions}} = \frac{{\text{MRO infection cases}}}{{\text{Total pa for the particular year}}} \times {1}00$$

#### Statistical analyses

Firstly, the Jonckheere-Terpstra test was performed to determine the trend of monthly antibiotic consumption, the proportion of pathogens and antimicrobial resistance rate over time. The J-T test is a non-parametric method to evaluate the presence of definite trend/order in the data. Separately, to evaluate the presence of linear relationship, Pearson’s correlation coefficient was used to investigate the association between antibiotic consumption and bacterial resistance rates. Statistical significance was defined at *p* < 0.05. Data analyses were conducted using Statistical Package for the Social Sciences (SPSS) version 28.0 (IBM Corporation, Armonk, NY).

#### Patient involvement

No patients were involved in this study. There was no plan to disseminate the results to the relevant patient community.

### Ethical approval

The study was reviewed and approved by the Medical Research and Ethics Committee of the Ministry of Health, Malaysia (NMRR-20-787-54141).

## Results

### Overall consumption and trends of parenteral antibiotics

Table 1 demonstrates the annual consumption of antimicrobials and their respective classes for parenteral use. The total consumption of six classes of antibiotics monitored was 167.49 DDD/100 admission for the 3-year study period. There was a 38.7% and 32.9% surge in antibiotic use in 2020 compared to 2018 and 2019 (146.456 DDD/100 admissions in 2018, 152.852 DDD/100 admissions in 2019 and 203.137 DDD/100 admissions in 2020, *p* < 0.001). Meanwhile, broad-spectrum antibiotics consumption increased by 37.6% and 31.7% in year 2020 from 2018 and 2019, respectively (127.061 DDD/100 admissions in 2018, 132.686 DDD/100 admissions in 2019 and 174.779 DDD/100 admissions in 2020, *p* < 0.001). Antibiotics against MRO showed a rising trend in 2020 by 46.2% and 40.6% from 2018 and 2019, respectively (19.396 DDD/100 admissions in 2018, 20.166 DDD/100 admissions in 2019 and 28.358 DDD/100 admissions in 2020, *p* = 0.001).

**Table 1:** Consumption of antimicrobial agents for parenteral use from 2018 to 2020 (DDD/100 admissions).

	2018	2019	2020	*p*-value*	Total
**Broad-spectrum antibiotics**					
**BL/BLIs**	**66.221**	**66.375**	**83.954**		**72.18 (43.1%)**
Amoxicillin-clavulanate	34.504	32.318	50.698	**0.008**	39.17 (23.4%)
Ampicillin-sulbactam	14.250	9.616	12.823	0.223	12.23 (7.3%)
Piperacillin-tazobactam	17.467	24.441	20.433	0.451	20.78 (12.4%)
**ESC**	**57.537**	**62.112**	**86.786**		**68.81 (41.1%)**
Ceftriaxone	40.030	43.128	54.572	**0.042**	45.91 (27.4%)
Cefotaxime	0.108	0.362	0.123	0.977	0.20 (0.1%)
Ceftazidime	6.071	6.778	7.429	0.104	6.76 (4.1%)
Cefoperazone	2.771	3.021	3.933	**0.010**	3.24 (1.9%)
Cefoperazone-sulbactam	0.299	0.584	0.378	0.954	0.42 (0.3%)
Cefepime	8.258	8.239	20.351	**0.005**	12.28 (7.3%)
**FQ**	**3.303**	**4.199**	**4.039**		**3.85 (2.2%)**
Ciprofloxacin	3.058	3.890	3.697	0.104	3.55 (2.0%)
Levofloxacin	0.245	0.309	0.342	0.826	0.30 (0.2%)
**Subtotal**	**127.061**	**132.686**	**174.779**	** < 0.001**	**144.84 (86.4%)**
**Antibiotics against MRO**					
**Carbapenems**	**11.012**	**11.873**	**17.530**		**13.47 (8.1%)**
Imipenem-cilastatin	1.170	2.343	1.042	0.674	1.52 (0.9%)
Meropenem	8.425	7.224	12.311	**0.005**	9.32 (5.6%)
Ertapenem	1.417	2.306	4.177	** < 0.001**	2.63 (1.6%)
**Polymyxins**	**2.085**	**2.053**	**2.680**		**2.28 (1.4%)**
Polymyxin B	0.008	0.004	0.281	0.354	0.10 (0.1%)
Colistin	2.077	2.049	2.399	0.653	2.18 (1.3%)
**Glycopeptide (**Vancomycin**)**	6.299	6.240	8.148	0.082	6.90 (4.1%)
**Subtotal**	**19.396**	**20.166**	**28.358**	0.001	**22.65 (13.6%)**
**Total**	**146.456**	**152.852**	**203.137**	< 0.001	**167.49 (100%)**

BL/BLI = beta-lactam/beta-lactamase inhibitors, ESC = extended spectrum cephalosporin, FQ = fluoroquinolones.

*Significant trend (J-T test on monthly data) at *p* < 0.05.

**Figure 1:**
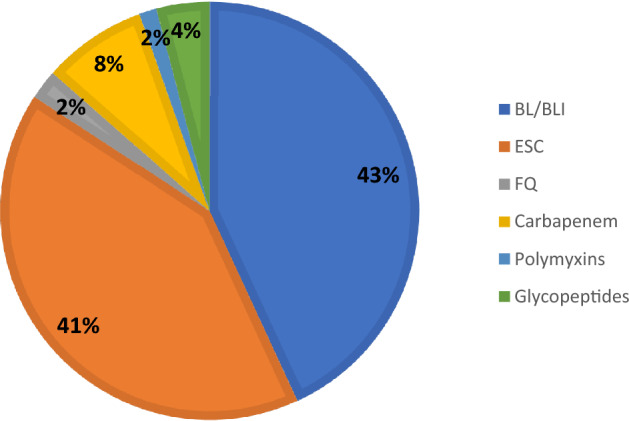
Consumption of antibiotics by subgroups from 2018 to 2020. BL/BLI = beta-lactam/beta-lactamase inhibitors, ESC = extended spectrum cephalosporins, FQ = fluoroquinolones.

Beta-lactam/beta-lactamase inhibitors (BL/BLIs) was the most frequently prescribed antibiotic groups (43.1%, 72.18/167.49 DDD/100 admissions), followed by ESC (41.1%,68.81/167.49 DDD/100 admissions), carbapenems (8.1%, 13.47/167.49 DDD/100 admissions), glycopeptides (4.1%, 6.90/167.49 DDD/100 admissions), FQ (2.2%, 3.85/167.49 DDD/100 admissions) and polymyxins (1.4%, 2.28/167.49 DDD/100 admissions) (Fig. 1, Table 1). The utilization of broad-spectrum antibiotics and antibiotics against MRO were 86.4% (144.84/167.49 DDD/100 admissions) and 13.6% (22.65/167.49 DDD/100 admissions), respectively.

The top five most frequently prescribed antibiotics, which accounted for 77.8% of all antibiotic usage, were amoxicillin-clavulanate, ampicillin-sulbactam, piperacillin-tazobactam, ceftriaxone and cefepime. All the antibiotics exhibited increasing use throughout the study period except for ampicillin-sulbactam and piperacillin-tazobactam, which demonstrated a dip in 2019 and 2020. Consumption of amoxicillin-clavulanate, ceftriaxone, cefoperazone, cefepime, meropenem and ertapenem was significantly greater (*p* < 0.05) throughout the 3-year observation.

**Figure 2:**
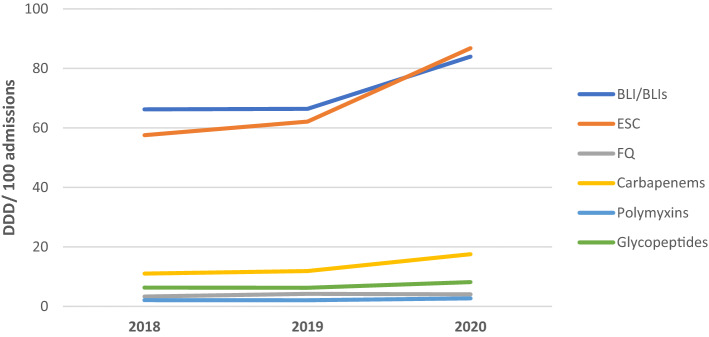
Annual consumption of different antibiotic classes from 2018 to 2020.

BL/BLI was the most used antibiotic group, with 26.8% and 26.5% increase in 2020 from 2018 and 2019, respectively (66.22 DDD/100 admissions in 2018, 66.375 DDD/100 admissions in 2019 and 83.954 DDD/100 admissions) (Table 1). On the other hand, the annual ESC usage revealed a 50.8% and 39.7% increase in 2020 from 2018 and 2019, respectively (57.537 DDD/100 admissions in 2018, 62.112 DDD/100 admissions in 2019 and 86.786 DDD/100 admissions in 2020). Figure 2 illustrates the antibiotic consumption based on their respective classes.

### Trends of MRO infection rates

**Figure 3:**
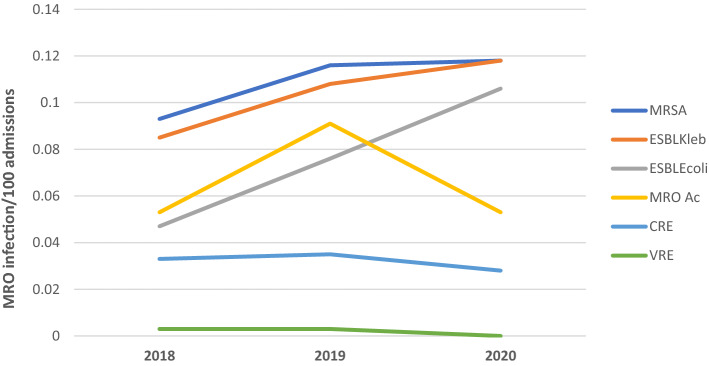
Trends of MRO infection cases per 100 admissions from 2018 to 2020.

The most isolated MRO pathogens between 2018 and 2020 was MRSA (28.0%), followed by ESBL-producing *Klebsiella spp.* (26.7%), ESBL-producing *E. coli* (19.6%), MRO *A. baumannii* (16.9%), CRE (8.2%) and VRE (0.5%) (Table 2). The relative infection rates of MRSA, ESBL *Klebsiella spp* and *ESBL E. coli* increased throughout the three years, whereas MRO*A. baumannii*, CRE and VRE infection rates decreased in 2020. Besides, *E. coli* presented significantly higher ESBL rates over the years (0.047 in 2018, 0.076 in 2019 and 0.106 in 2020, *p* = 0.004). Similarly, the VRE rate was significantly lower in 2020 compared to 2019 and 2018 (0.003 in 2018 and 2019 and 0.000 in 2020, *p* = 0.042). Figure 3 demonstrates the antibiotic resistance pathogens observed for three years.

**Table 2:** Rate of MRO infection per 100 admissions from 2018 to 2020.

Organisms	2018	2019	2020	*p*-value*	Total (%)
Methicillin-resistant *Staphylococcus aureus* (MRSA)	0.093	0.116	0.118	0.281	0.11 (28.0%)
Extended spectrum beta-lactamase (ESBL)- producing *Klebsiella* spp	0.085	0.108	0.118	0.056	0.10 (26.7%)
Extended spectrum beta-lactamase (ESBL)- producing *Escherichia coli*	0.047	0.076	0.106	**0.004**	0.08 (19.6%)
Multidrug-resistant organisms (MRO) *Acinetobacter baumannii*	0.053	0.091	0.053	0.919	0.07 (16.9%)
Carbapenem-resistant Enterobacteriaceae (CRE)	0.033	0.035	0.028	0.554	0.03 (8.2%)
Vancomycin- resistant enterococci (VRE)	0.003	0.003	0.000	**0.042**	0.00 (0.5%)

*Significant trend (J-T test on monthly data) at *p* < 0.05.

### Correlation between antibiotic consumption and MRO infection rates

The correlation between antibiotic consumption and MRO occurrence is summarised in Table 3.

**Table 3:** Correlation between antibiotic consumption and MRO occurrence.

Organism	Antibiotic group	Correlation	
		*p-*value	Correlation coefficient (Pearson’s *r*)
MRSA	BL/BLIs	0.683	− 0.071
	ESC	**0.011**	0.421*
	FQ	**0.001**	0.541**
	Carbapenem	0.310	0.174
	Polymyxins	0.753	− 0.054
	Glycopeptides	0.430	0.136
ESBL- producing *Klebsiella* spp	BL/BLIs	0.442	− 0.132
	ESC	** < 0.001**	0.627**
	FQ	**0.023**	0.377*
	Carbapenem	**0.016**	0.400*
	Polymyxins	**0.033**	0.356*
	Glycopeptides	0.522	0.110
ESBL- producing *E. coli*	BL/BLIs	0.529	0.108
	ESC	**0.001**	0.524**
	FQ	**0.002**	0.503**
	Carbapenem	**0.009**	0.431**
	Polymyxins	0.446	0.131
	Glycopeptides	0.099	0.279
MRO *A. baumannii*	BL/BLIs	**0.030**	− 0.362*
	ESC	**0.025**	0.373*
	FQ	**0.003**	0.479**
	Carbapenem	0.225	0.207
	Polymyxins	**0.033**	0.356*
	Glycopeptides	0.742	0.057
CRE	BL/BLIs	**0.039**	− 0.346*
	ESC	0.100	0.279
	FQ	0.054	0.324
	Carbapenem	0.404	0.144
	Polymyxins	**0.038**	0.348*
	Glycopeptides	0.655	0.077
VRE	BL/BLIs	0.735	− 0.058
	ESC	0.160	− 0.239
	FQ	0.152	− 0.244
	Carbapenem	0.128	− 0.258
	Polymyxins	0.198	− 0.220
	Glycopeptides	**0.031**	− 0.359*

BL/BLIs = beta lactam/beta-lactamase inhibitors, ESC = extended-spectrum cephalosporin (third generation and fourth generation cephalosporins), FQ = fluoroquinolones.

Significant result *p < 0.05, **p < 0.01.

Increasing BL/BLI consumption demonstrated significant negative correlation for occurrence of MRO *A. baumannii* (r =  − 0.362, *p* = 0.03) and CRE (r =  − 0.346, *p* = 0.039). The consumption of ESC resulted in significant positive correlation for occurrence of MRSA (r = 0.421, *p* = 0.011), ESBL-producing *Klebsiella* spp (r = 0.627, *p* =  < 0.001), ESBL-producing *E. coli* (r = 0.524, *p* = 0.001) and MRO *A. baumannii* (r = 0.373, *p* = 0.025). Similarly, FQ showed significant positive correlation for occurrence of MRSA (r = 0.541, *p* = 0.001), ESBL-producing *Klebsiella* spp (r = 0.377, *p* = 0.023), ESBL-producing *E. coli* (r = 0.503, *p* = 0.002) and MRO (r = 0.479, *p* = 0.003). Carbapenem demonstrated significant positive correlation with ESBL-*Klebsiella* spp (r = 0.400, *p* = 0.016) and ESBL-*E. coli* (r = 0.431, *p* = 0.009). In comparison, polymyxins were associated with significant positive correlation for resistance of ESBL-producing *Klebsiella* spp (r = 0.356, *p* = 0.033), MRO *A. baumannii* (r = 0.356, *p* = 0.033) and CRE (r = 0.348, *p* = 0.038). VRE occurrence negatively correlated with the consumption of glycopeptides (r =  − 0.359, *p* = 0.031).

## Discussion

The study reported the antibiotic usage and antimicrobial resistance patterns in the biggest tertiary hospital in Kuala Lumpur, Malaysia. Antibiotic Point Prevalent Survey of HKL in 2018 revealed that 88.5% of antibiotics prescribed was for empirical therapies^16^. Jamaluddin et al*.* reported that empirical antimicrobials accounted for approximately 65% of prescribed antibiotics, notably throughout medical, surgical, orthopaedic and oncology wards in Malaysia^17^.The initial antimicrobial therapy is based on clinical judgments, emphasising likely pathogens while awaiting laboratory reports. Ideally, causative microorganisms’ identification enables the clinicians to select the most appropriate antibiotic, which improves overall patient outcomes^18^. Nevertheless, the culture and sensitivity results generally take more than 48 h to be available. Hence, the clinicians had to rely on the most likely organisms and site of infections for antibiotic selection^17^.

In this study, the most consumed antibiotics for hospitalised patients were broad-spectrum antibiotics subgroups, ESC and BL/BLI, comprising 86.4% of total antibiotic consumption. These results are comparable across Ministry of Health (MOH) hospitals, university hospitals, military hospitals, and private hospitals in Malaysia^14^. Similarly, the reports from the Global Point prevalence study (PPS) revealed BLI and ESC are among the top three antibiotics prescribed worldwide^18^. Furthermore, the total utilisation of BL/BLI and ESC increased steadily every year, where a substantial increase was evident from 2019 to 2020 by 26.5% and 39.7%, respectively. Meanwhile, a significant rise in carbapenem, mainly ertapenem, was observed in this study. This might be related to the efforts of de-escalating broad-spectrum carbapenem such as imipenem or meropenem to ertapenem in ESBL infection. However, meropenem consumption increased steadily over the years as a preferred choice of empirical carbapenem due to its safety profile and convenient dosing.

Implementation of Protocol for Antimicrobial Stewardship (AMS) by MOH in 2014 was a successful approach to reduce unnecessary and inappropriate use of antibiotics, resulting in the decreasing trend of total antibiotic consumption in MOH hospitals^13^. However, there was a hike in HKL’s entire antibiotic consumption trend, especially in 2020. Given that, Haug et al*.* suggested a few factors that can influence the variability in antibiotic consumption, including higher nurse staffing and larger proportions of shorter (< 2 days) and longer (> 10 days) hospital stays can lead to an increased antibiotic use^19^. Moreover, variations in local antimicrobial resistance patterns, physicians’ prescribing behaviour and workloads can also lead to discrepancies in antibiotic consumption^20,21^. Therefore, it is essential to consider these factors when comparing antibiotic utilisation with the national reports.

MRO is one of the most crucial threats to national healthcare, typically associated with nosocomial infections. The indiscriminate and overuse of antibiotics, especially the broad-spectrum category, are the major factors associated with rising bacterial resistance. Studies by Meyer et al*.* and Wushouer et al*.* found a significant correlation between broad-spectrum antibiotics use and MRO^3,22^. Furthermore, Barnes et al*.* concluded that the reduction in antibiotic use significantly reduces the rate of MRO occurrence^23^. In the present study, MRSA and ESBLs showed increased resistance patterns throughout the 3-year observation period. The increase in infection rate may result from high usage of certain antibiotic groups such as FQ and ESC and possibly poor infection control. A major part of healthcare-associated infections is avoidable through effective infection prevention and control strategy that will substantially contain antimicrobial resistance and outbreaks of highly transmissible diseases, including the COVID-19 pandemic. It is evident that infection rate can be contained by establishing local and national standard operating procedures (SOP), especially on five major components that include: (i) modification of system to facilitate good practice, (ii) training and educating healthcare personnel, (iii) observing health practices, procedures and outcomes and delivering timely feedback, (iv) refining healthcare message, and (v) adapting to a safety climate^24^. A study by Nori et al*.* on SARS-CoV-2 identified the most common opportunistic pathogens were *S. aureus* (44%), *P. aeruginosa* (16%), *Klebsiella* spp. (10%), *Enterobacter.* spp. (8%) and *E. coli* (4%) while MRO isolates were present in 15% of the testing^25^. The emergence of MRO CRE poses challenges to healthcare institutions globally, including Malaysia, since carbapenem is one of the ‘last-line antimicrobials where treatment options are limited, and higher mortality rates are reported compared to other MRO strains^26^. Moreover, the review of carbapenem at 72 h following AMS is believed to assist in carbapenems de-escalation to substantially reduce the risk of CRE^27^.

Antibiotic resistance directly influences antimicrobial prescription by increasing the burden by mitigating certain classes of antibiotic class to another to reduce the selective pressure towards a particular antimicrobial class. Based on the current investigation, ESC and FQ had a stronger association with MRO than other antibiotic groups, including MRSA, ESBLs and MRO *A. baumannii*. Several studies described that ESC-resistant isolates were associated with gram-negative microorganisms’ production of ESBL, plasmid-mediated AmpCs, carbapenemase enzymes and MRSA occurrence^28,29^. In addition, Wushouer et al*.* reported significant positive correlations between MRSA infection rates and the consumption of third generation cephalosporins, carbapenems and glycopeptides^22^.

FQ consumption contributed to the prevalence of ESBL-producing pathogens, consistent with a surveillance study in China^30^. Ryu et al*.* explained that the positive correlation between FQ and *Klebsiella* spp was related to the transferable plasmid (quinolone-resistant (qnr) gene) in ESBL-producing strains that lead to FQ resistance^31^. *Klebsiella* spp, especially *K. pneumoniae,* adapts well in the hospital environment and survives longer than other Enterobacteriaceae; thus, allowing for easier cross-infection, which deems for stricter control of the antibiotics in the future^32^. In the current study, carbapenem use showed a positive correlation with ESBL infections despite being used in the AMS strategy as the definitive treatment for ESBLs. Martìnez et al*.* reported that previous carbapenem use was a significant risk factor for ESBL-*Klebsiella* spp. and *E. coli* based on the possibilities of antibiotic enriching the gut reservoir with ESBL in patients already being colonised with a low number of microbes or promoting bacterial settlement following transmission event^33^. Besides, the positive correlation between polymyxins and ESBL-*Klebsiella* spp, MRO *A. baumannii,* and CRE may be linked to the chromosomal mutations of the carbapenem-resistant *A. baumannii* ST1 and CrrB single nucleotide polymorphism (SNP) mutants of polymyxins^34,35^.

The negative incidence of VRE to glycopeptides in the study may be linked to the comprehensive strategies that include appropriate dosing and duration of administration^24^. Interestingly, a negative correlation between BL/BLI and MRO *A. Baumannii* and CRE may have been contributed by reversed bacterial resistance mechanisms based on the antibiotic’s preferential targeting of specific cell wall assembly machinery^36^.

In order to curb the increasing antibiotic consumption trend, we have introduced several actions plans to improve antibiotic usage in our setting, including strict implementation of antibiotic request forms and antibiotic stop orders. The prescribers are required to submit an antibiotic request form upfront when prescribing six types of antibiotics, including meropenem, imipenem, ertapenem, vancomycin, colistin and polymyxin B. An antibiotic stop order is activated if these antibiotics are continued beyond 72 h, without any justification or evidence from culture and sensitivity results. In addition, our setting also embarked on persuasive antimicrobial stewardship initiatives such as education to all healthcare professionals through antimicrobial awareness week events and antimicrobial stewardship grand ward rounds.

### Limitations

The study focused solely on the prevalence of antibiotic usage, primarily due to the nature of the cross-sectional study design. Other factors that could potentially influence antimicrobial use include the COVID-19 outbreak in 2020 and the patient case-mix. Therefore, the retrospective and observational study design cannot confirm the causal relationship between antibiotics consumption and MROs infection rates. Nevertheless, there was a positive correlation between the two variables. Therefore, the findings can suggest ways to reduce antimicrobial resistance. Furthermore, due to the different dosage requirements, antibiotic consumption was measured using DDD, which may be inaccurate and underestimates certain patient groups such as renal impairment. Therefore, it may not represent the prescribed daily dose for all patients.

Moreover, there was a lack of patient-specific data for detailed risk factor evaluation. Since certain patients with a higher risk of MRO infection at baseline or cross-infection of MROs may affect the statistical significance, future research should include demographic data to address this gap. In addition, more data points (eg.10-year trends) should be considered to prove the correlation between antibiotics’ consumption and MROs infection.

## Conclusion

The overall data presented a fair indicator for antibiotic use and resistance pattern in a tertiary hospital in Malaysia. Broad-spectrum antibiotics and antibiotics against MRO consumption increased significantly throughout the study, while the antimicrobial resistance trend in the hospital decreased, except for MRSA and ESBL. In addition, ESC and FQ consumption was positively correlated with the emergence of MRSA, ESBL-producing *Klebsiella* spp, ESBL-producing *E. coli* and MRO *A. baumannii* resistance. Continuous review and reinforcement of AMS in tertiary care settings are thus crucial to overcome the potential threats by MROs.

## References

[1] Gould IM, Bal AM (2013). New antibiotic agents in the pipeline and how they can help overcome microbial resistance. Virulence.

[2] World Health Organization. *Global Antimicrobial Resistance Surveillance System (GLASS) Report: Early Implementation 2017–2018.* (World Health Organization, 2018).

[3] Meyer E, Gastmeier P, Deja M, Schwab F (2013). Antibiotic consumption and resistance: data from Europe and Germany. Int. J. Med. Microbiol..

[4] Dhingra S (2020). Microbial resistance movements: an overview of global public health threats posed by antimicrobial resistance, and how best to counter. Front. Publ. Health.

[5] Chandy SJ, Naik GS, Balaji V, Jeyaseelan V, Thomas K, Lundborg CS (2014). High cost of burden and health consequences of antibiotic resistance: the price to pay. J. Infect. Dev. Ctries..

[6] Kim CJ (2014). The burden of nosocomial Staphylococcus aureus bloodstream infection in South Korea: a prospective hospital-based nationwide study. BMC Infect Dis..

[7] Haque M, Sartelli M, McKimm J, Abu Bakar M (2018). Health care-associated infections – an overview. Infect. Drug Resist..

[8] Fair RJ, Tor Y (2014). Antibiotics and bacterial resistance in the 21^st^ century. Perspect. Medicin. Chem..

[9] Ventola CL (2015). The antibiotic resistance crisis. Pharm. Ther..

[10] Sabtu N, Enoch DA, Brown NM (2015). Antibiotic resistance: what, why, where, when and how?. Br. Med. Bull..

[11] National center for Emerging and Zoonotic Infectious Disease. *Core elements of hospital antibiotic stewardship programs.* (Centres for Disease control and Prevention, 2019). 10.1093/cid/ciu542 PMC6521960 25261548

[12] Ministry of Health Malaysia. *Protocol on Antimicrobial Stewardship Program in Healthcare Facilities.* 1st Ed. (Ministry of Health Malaysia, 2014).

[13] Pharmaceutical Services Programme. *National Antimicrobial Guideline.* (Ministry of Health Malaysia, 2019).

[14] Ministry of Health Malaysia. *Malaysian Action Plan on Antimicrobial Resistance (MyAP-AMR) 2017–2021.* (Ministry of Health, and Ministry of Agriculture and Agro-Based Industry Malaysia, 2017).

[15] World Health Organization. *ATC-DDD Toolkit - DDD Indicators: Introduction to DDD Indicators*. (World Health Organization, 2022).

[16] Hospital Kuala Lumpur Infection Control and Antibiotic Committee on Antibiotic Point Prevalence Survey. (Hospital Kuala Lumpur, 2018).

[17] Jamaluddin NAH (2021). Point prevalence survey of antimicrobial use in a Malaysian Tertiary Care University Hospital. Antibiotics.

[18] Versporten A (2018). Antimicrobial consumption and resistance in adult hospital inpatients in 53 countries: results of an internet-based global point prevalence survey. Lancet Glob. Health.

[19] Haug JB, Berild D, Walberg M, Reikvam Å (2014). Hospital- and patient-related factors associated with differences in hospital antibiotic use: analysis of national surveillance results. Antimicrob. Resist. Infect. Control.

[20] Calbo W, Alvarez-Rocha L, Gudiol F, Pasquau J (2013). A review of the factors influencing antimicrobial prescribing. Enferm. Infecc. Microbiol. Clin..

[21] Chem ED, Anong DN, Akoachere JKT (2018). Prescribing patterns and associated factors of antibiotic prescription in primary health care facilities of Kumbo East and Kumbo West Health Districts. Northwest Cameroon. PLoS One.

[22] Wushouer H (2018). Trends and relationship between antimicrobial resistance and antibiotic use in Xinjiang Uyghur Autonomous Region, China: Based on a 3-year surveillance data, 2014–2016. J. Infect. Publ. Health.

[23] Barnes SL, Rock C, Harris AD, Cosgrove SE, Morgan DJ, Thom KA (2017). The impact of reducing antibiotics on the transmission of multidrug-resistant organisms. Infect. Control Hosp. Epidemiol..

[24] Storr J (2017). Core components for effective infection prevention and control programmes: new WHO evidence-based recommendations. Antimicrob. Resist. Infect. Control.

[25] Nori P (2021). Bacterial and fungal coinfections in COVID-19 patients hospitalized during the New York City pandemic surge. Infect. Control Hosp. Epidemiol..

[26] Zaidah AR, Mohammad NI, Suraiya S, Harun A (2017). High burden of Carbapenem-resistant Enterobacteriaceae (CRE) fecal carriage at a teaching hospital: cost-effectiveness of screening in low-resource settings. Antimicrob. Resist. Infect. Control.

[27] Mani NS (2020). Post-prescription review with threat of infectious disease consultation and sustained reduction in meropenem use over four years. Clin. Infect. Dis..

[28] Seiffert SN, Hilty M, Perreten V, Endimiani A (2013). Extended-spectrum cephalosporin-resistant Gram-negative organisms in livestock: an emerging problem for human health?. Drug Resist. Updat..

[29] Upreti N, Rayamajhee B, Sherchan S, Choudhari MK, Banjara MR (2018). Prevalence of methicillin resistant Staphylococcus aureus, multidrug resistant and extended spectrum β-lactamase producing gram negative bacilli causing wound infections at a tertiary care hospital of Nepal. Antimicrob. Resist. Infect. Control.

[30] Wang R, Yang Q, Zhang S, Hong Y, Zhang MH, Jiang S (2019). Trends and correlation of antibiotic susceptibility and antibiotic consumption at a large teaching hospital in China (2007–2016): a surveillance study. Ther. Clin. Risk Manag..

[31] Ryu S, Klein EY, Chun BC (2018). Temporal association between antibiotic use and resistance in Klebsiella pneumoniae at a tertiary care hospital. Antimicrob. Resist. Infect. Control.

[32] van der Steen, M., Leenstra, T., Kluytmans, J. A., van der Bij, A. K. & ISIS-AR study group. Trends in Expanded-Spectrum Cephalosporin-Resistant Escherichia coli and Klebsiella pneumoniae among Dutch Clinical Isolates, from 2008 to 2012. *PLoS One* **10**(9):e0138088 (2015). 10.1371/journal.pone.0138088 PMC4575062 26381746

[33] Martínez JA (2006). Prior use of carbapenems may be a significant risk factor for extended-spectrum β-lactamase-producing *Escherichia coli* or *Klebsiella* spp. in patients with bacteraemia. J. Antimicrob. Chemother..

[34] Carrasco LDM (2021). Polymyxin Resistance Among XDR ST1 Carbapenem-Resistant *Acinetobacterbaumannii* Clone Expanding in a Teaching Hospital. Front. Microbiol..

[35] McConville TH (2020). CrrB Positively Regulates High-Level Polymyxin Resistance and Virulence in *Klebsiella pneumoniae*. Cell Rep..

[36] Geisinger E (2020). Antibiotic susceptibility signatures identify potential antimicrobial targets in the *Acinetobacter baumannii* cell envelope. Nat. Commun..

